# Sensitivity Analysis of Fatigue Crack Growth Model for API Steels in Gaseous Hydrogen

**DOI:** 10.6028/jres.119.002

**Published:** 2014-02-25

**Authors:** Robert L Amaro, Neha Rustagi, Elizabeth S Drexler, Andrew J Slifka

**Affiliations:** National Institute of Standards and Technology, Boulder, CO 80305

**Keywords:** API steel, hydrogen-assisted fatigue crack growth, model, sensitivity analysis

## Abstract

A model to predict fatigue crack growth of API pipeline steels in high pressure gaseous hydrogen has been developed and is presented elsewhere. The model currently has several parameters that must be calibrated for each pipeline steel of interest. This work provides a sensitivity analysis of the model parameters in order to provide (a) insight to the underlying mathematical and mechanistic aspects of the model, and (b) guidance for model calibration of other API steels.

## 1. Introduction

Hydrogen is expected to help bridge the gap between an energy infrastructure based primarily upon fossil fuels and one dominated by renewable resources. A key step towards the utilization of hydrogen as an energy carrier is a hydrogen transportation infrastructure. In order to ensure a safe hydrogen transportation system, engineering models must be developed that can help pipeline design engineers and operators determine safe operating conditions and life prediction for the pipeline materials of interest. A model to predict hydrogen-assisted (HA) fatigue crack growth (FCG) of API pipeline steels has been proposed. The detailed background of the model derivation and full calibration for API X100 steel can be found in [1], while successful partial calibration of the model for API X100 and X52 steels by use of a reduced number of upfront tests can be found in [2]. Additionally, the constitutive model supporting the HA FCG model, HA FCG predictive model implementation in MATLAB, and application of the overall model to predict pressure vessel cycles to failure are detailed in [3]. The model has been shown to perform well at predicting HA FCG of pipeline steels as a function of operating pressure, and FCG driving force (*ΔK*).

The total HA FCG is predicted by

$${\frac{da}{dN}}_{total}={\frac{da}{dN}}_{fatigue}+\delta (P_{H}-P_{H_{th}}){\frac{da}{dN}}_{H},$$ (1)

where ${\frac{da}{dN}}_{fatigue}$ is the contribution to the crack extension per cycle (*da/dN*) from fatigue in air and ${\frac{da}{dN}}_{H}$ the FCG contribution from fatigue in gaseous hydrogen. The delta operator represents the Heaviside step function, which produces a value of zero value when the term in the parenthesis is zero or negative, and a value of one otherwise. *P_H_* is the hydrogen operating pressure of interest and $P_{H_{th}}$ threshold hydrogen pressure below which HA FCG does not occur. The Heaviside step function enables the model to predict FCG in air, when the hydrogen pressure is sufficiently low, and also predict HA FCG when $P_{H}>P_{H_{th}}$. Fatigue crack growth in air is given by

$${\frac{da}{dN}}_{fatigue}=A\Delta K^{b},$$ (2)

where *A* and *b* are material specific constants and *ΔK* is the range of the stress intensity factor. The HA FCG is predicted with a cumulative damage methodology:

$${\frac{da}{dN}}_{H}={\left[{\left({\frac{da}{dN}}_{P_{H}}\right)}^{-1}+{\left({\frac{da}{dN}}_{\Delta K}\right)}^{-1}\right]}^{-1},$$ (3)

where ${\frac{da}{dN}}_{P_{H}}$ is the HA FCG resulting from the hydrogen-dominated material response (transient regime) and ${\frac{da}{dN}}_{\Delta K}$ is the HA FCG resulting from the *ΔK*-dominated material response (steady state regime). The first term is defined as

$${\frac{da}{dN}}_{P_{H}}=a1\Delta K^{B1}{\left(P_{H}^{m1}\exp\left(\frac{-Q+V\sigma_{h}}{RT}\right)\right)}^{d1},$$ (4)

and the second term is defined as

$${\frac{da}{dN}}_{\Delta K}=a2\Delta K^{B2}{\left(P_{H}^{m2}\exp\left(\frac{-Q+V\sigma_{h}}{RT}\right)\right)}^{d2}.$$ (5)

The variables *a1*, *B1*, *m1*, *d1*, *a2*, *B2*, *m2*, and *d2* are all dimensionless constants; *Q* is the activation energy for hydrogen concentration in steel (*Q*=27.1 kJ/mole-K [4]); *V* is the partial molar volume of hydrogen in the metal (*V*=2.0 × 10^−6^ m^3^/mol [5]); *R* is the universal gas constant; *T* is the absolute temperature; and *σ*_ℎ_ is the hydrostatic stress at the crack tip defined as

$$\sigma_{h}=\frac{1}{3}\sigma_{ii},$$ (6)

where *σ_ii_* is the trace of the stress tensor. The model is based upon the assumption that for low *ΔK*, and therefore low crack extension per cycle, the HA FCG is dominated by the stress-assisted hydrogen concentration very near the crack tip. This region is highly influenced by the fatigue process zone (FPZ). This FCG regime is termed the transient regime and is described by Eq. (4). For larger *ΔK*, the associated fatigue crack extension per cycle extends beyond a critical distance, termed the transition crack length. The transition crack length is proportional to the plane strain FPZ size (*r_FPZ_*), estimated by [6]

$$r_{FPZ}=\frac{1}{6\pi }{\left(\frac{K_{max}}{\sigma_{Y}}\right)}^{2},$$ (7)

where *K_max_* is the maximum stress intensity factor and *σ_Y_* is the material yield stress. When the per-cycle FCG extends beyond the increased stress-assisted hydrogen concentration near the crack tip, the HA FCG rate decreases, and the resultant *da/dN* vs. *ΔK* Paris slope approaches that of air. This FCG regime is termed the steady state regime and is given by Eq. (5). The parameter values for the full model calibration to an API X100 steel are provided in Table 1.

In its current form the HA FCG model is purely phenomenological. Future research includes examination of the effects of microstructure on the FCG response, thereby providing more insight into the relative difference in model parameter values for calibration to different materials. Furthermore, research which examines the sub fracture surface dislocation structure is proposed to de-convolute the effect of the stress-assisted hydrogen concentration interactions with the per-cycle crack advance. The first step towards understanding how the model correlates the presumed microstructure and hydrogen concentration-to-crack tip advance interactions is accomplished by performing a sensitivity analysis of the model parameters controlling these aspects. Given that one must fit these model parameters to experimental results in order to calibrate the model, a sensitivity analysis will provide guidance as to how to perform the calibration.

## 2. Model Parameter Sensitivity Analysis

A sensitivity analysis of the parameters *B1*, *B2*, *m1*, and *m2* was conducted to assess the response of the predictive model outlined above. The baseline model parameters are provided in Table 1 and are from full calibration to an API 5L X100 pipeline steel. The model separates hydrogen-assisted crack growth into two regimes: transient and steady state. Crack growth within each regime is modeled as being controlled by different FCG mechanisms. The transient FCG regime incorporates the exponents *B1* and *m1*, whereas the steady-state FCG regime incorporates the exponents *B2* and *m2*, to produce predictive FCG trends. The parameters *B1* and *B2* are the exponents on *ΔK* in the transient and steady-state regimes, respectively, while *m1* and *m2* are exponents on the hydrogen pressure term in the transient and steady-state regimes, respectively. Looking at Eqs. (4) and (5), one notices that the ambient hydrogen pressure in both regimes is raised to the product of *mi* and *di*, where *i*=1 or *i*=2. The sensitivity analysis of the pressure exponent was conducted by varying only *m1* and *m2* while keeping *d1* and *d2* constant.

**Fig. 1:**
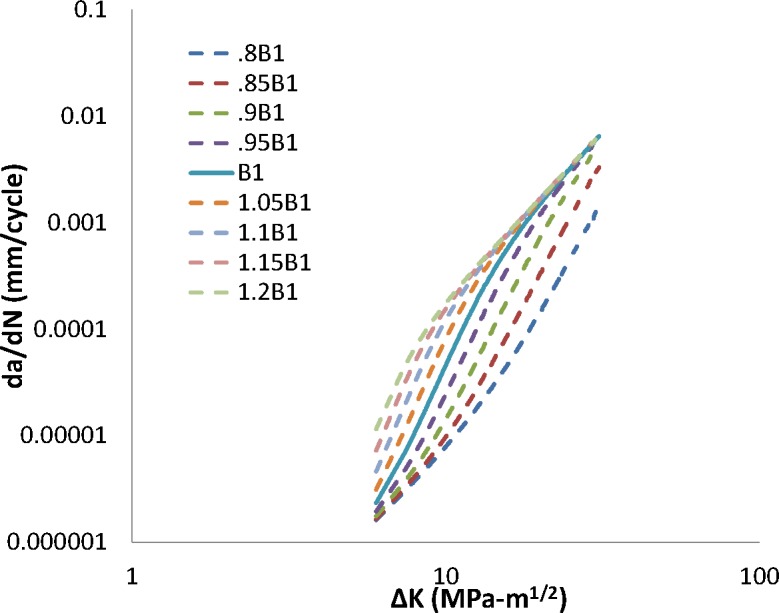
Impact of change in B1 on crack growth rate.

**Fig. 2:**
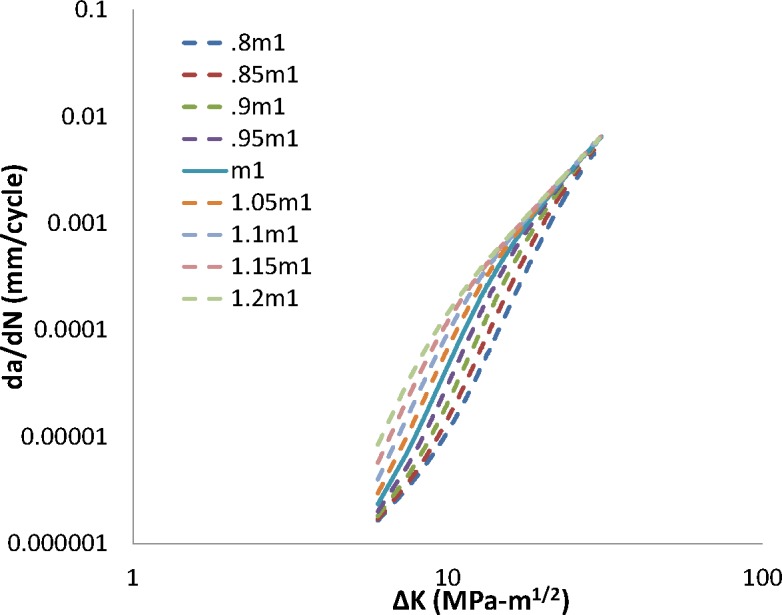
Impact of change in m1 on crack growth rate.

The transient FCG regime is dominated by interactions between the per-cycle crack extension and the stress-assisted hydrogen concentration within the FPZ. Results of a variation of the transient-regime input parameters by ± 20 % are shown in Figs. 1 and 2.

An increase in the exponents *B1* and *m1* yields an increase in the FCG rate in the transient regime. Moreover, the model responds similarly when increasing either parameter, indicating a strong coupling between these parameters at values larger than the baseline parameter value. As either parameter is decreased, the transient portions of the FCG curves approach the trend of FCG in air, as would be expected. However, a *decrease* in the exponent on *ΔK* (that is *B1*) has a far greater effect on the predicted FCG than an identical *decrease* in the exponent *m1* on hydrogen pressure. The parameter *B1* consequently has a larger impact than *m1* on the predicted transition-to-steady-state FCG, as well as the predicted steady-state FCG response. This can be seen graphically by the trend in *da/dN_tr_* shown in Figs. 1 and 2. In general, for the baseline parametric values studied in the transient regime of FCG, a change in the hydrogen pressure dependence via a modification in the exponent *m1* shifts the predicted transient FCG curve left or right, while holding the transition crack growth rate (which is equal to the transition crack length) relatively stationary. Modifying *B1*, on the other hand, greatly modifies the predicted transition crack growth rate. The transition crack growth rate increases as the model’s dependence on *ΔK* is decreased.

**Fig. 3:**
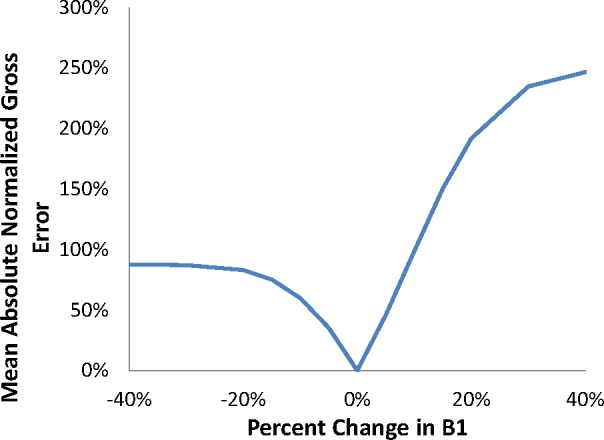
MANGE due to changes in B1.

**Fig. 4:**
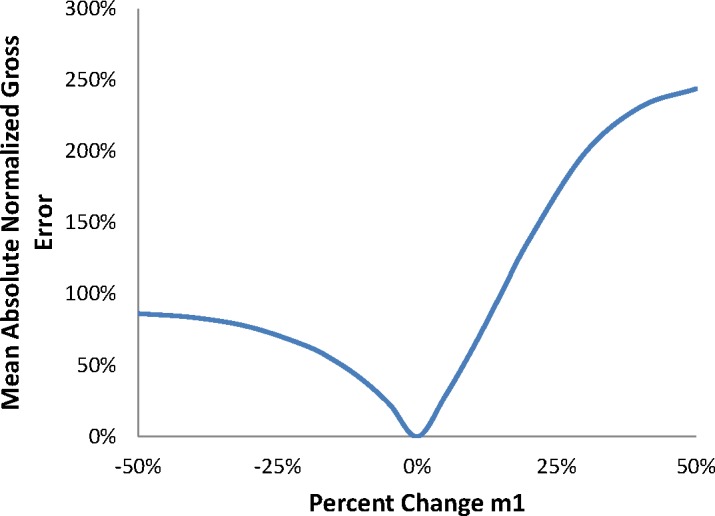
MANGE due to changes in m1.

The Mean Absolute Normalized Gross Error (MANGE) approach [7] can be used to determine the “accuracy” of a model that predicts a data set (rather than a single value), if a “correct” set of values is known. Specifically, the MANGE quantifies the average absolute value of the residuals (in percent) and is determined by

$$\text{MANGE}=\frac{1}{N}\sum_{i=1}^{N}\left|x_{i}-y_{i}\right|\times 100\%,$$ (8)

where *N* is the total number of samples being analiyzed in the data set, *x_i_* is the predicted value of a sample, and *y_i_* is the reference value (frequently an observation or known quantity). Whereas the MANGE is typically used to quantify an error, it was used in this analysis to assess the overall response of the model predictions to deviations in pressure and *ΔK* dependence. A single MANGE value was calculated from the data set produced from each value of the particular independent variable of interest. The model predictions resulting from the baseline parameter values are assigned to *y_i_*, and the model predictions produced from a change in parameter value are assigned to *x_i_*. As an example, one MANGE value was calculated from the data set with 0.8·*m1*, another MANGE value was calculated from the data set with 0.85·*m1*, and so on. As the deviation in independent variables is reduced, the nexus of the resulting MANGE values becomes a line. Figures 3 and 4 provide the resulting MANGE values as a function of the model parameter, *B1* and *m1*, respectively.

Figures 3 and 4 indicate that the model response approaches an asymptote at reductions of approximately 30 % in *B1* and 50 % in *m1*. Furthermore, the model response nearly saturates at increases of approximately 40 % in *B1* and 50 % in *m1*. While the model responds similarly to a decrease in either *B1* or *m1*, the rate of the model response is greater for an increase in *B1*, as opposed to an increase in *m1*. In general, modification of the exponent on *ΔK* has greater impact upon the model response within the transient FCG regime.

**Fig. 5:**
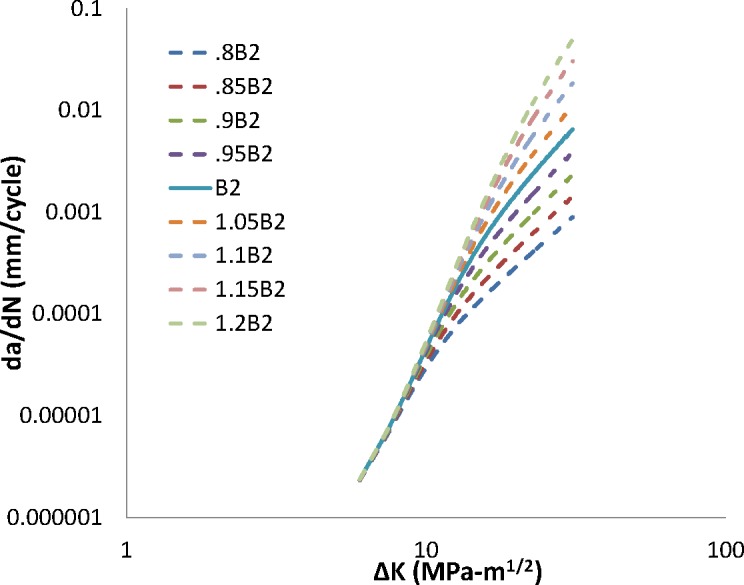
Impact of change in B2 on crack growth rate.

**Fig. 6:**
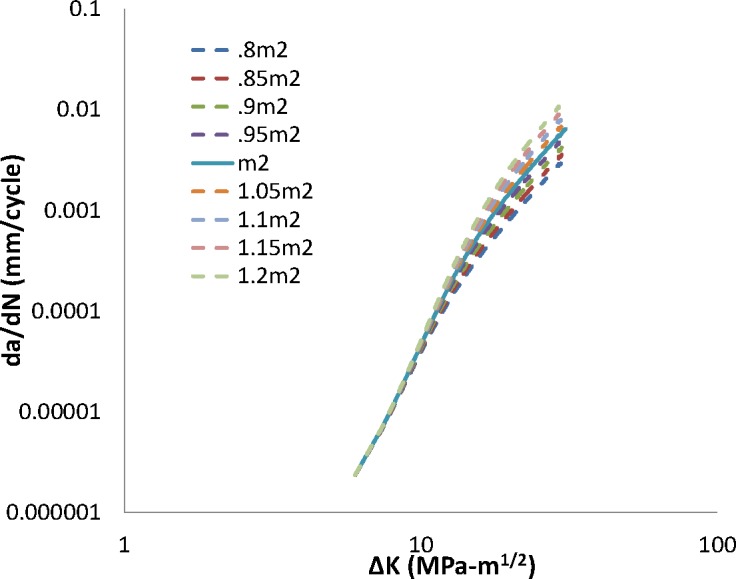
Impact of change in m2 on crack growth rate.

Steady-state FCG is presumed to be dominated by interactions between the fatigue crack extension and the far-field hydrogen concentration within the material (*i.e*., the hydrogen concentration beyond the FPZ). Model response in this regime is affected by the exponents *B2* and *m2* acting upon *ΔK* and the hydrogen pressure, respectively. Model response as a variation of *B2* is shown in Fig. 5, while model response as a variation of *m2* is shown in Fig. 6.

**Fig. 7:**
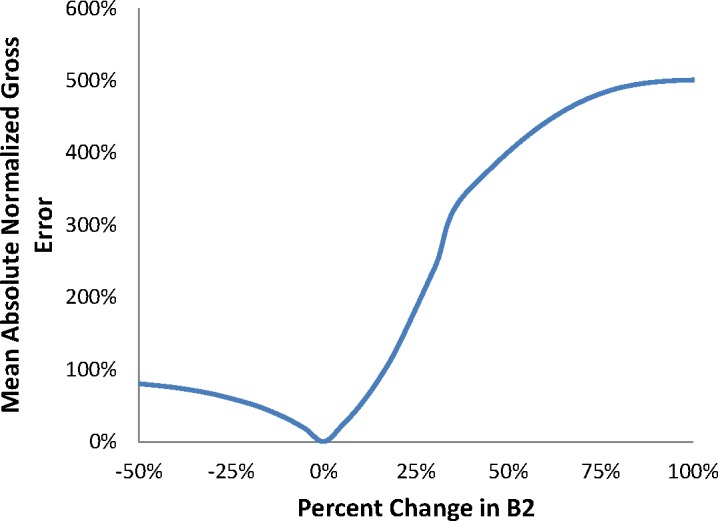
MANGE due to changes in B2.

**Fig. 8:**
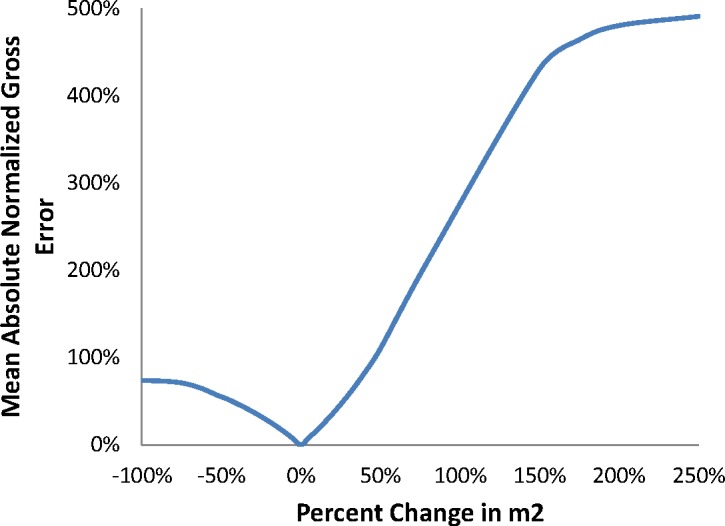
MANGE due to changes in m2.

Increasing either *B2* or *m2* increases model crack growth rate predictions as well as the transition crack length. Decreasing either parameter value does the converse. As in the transient regime, changing the *ΔK* dependence by modifying *B2* has a far greater effect on the predicted crack growth rate and transition crack length than changing the pressure dependence, *m2*. The impact of a change in *B2* or *m2* is negligible at low values of *ΔK* and becomes increasingly large in the steady-state regime, as expected from the cumulative damage formulation of the model. Results of MANGE analysis as a function of modifications of the parameters *B2* and *m2* are shown graphically in Figs. 7 and 8, respectively.

As in the transient regime, the MANGE analysis indicates that the model response plateaus at similar final states for both increases and decreases in either *B2* or *m2*. Modification to the variable *B2* has greater impact upon model response for small deviations from the baseline parameter values.

Determining “correct” values for the four parameters studied (*B1*, *m1*, *B2*, and *m2*) is impossible at this point in the research; however, determining the appropriate bounds for the variable is possible. As an example, the lower bound for *m1* and *m2* is zero, or a non-existent hydrogen pressure dependence, (*i.e*., that of air). Similarly, the lower bound of *B1* and *B2* is that of air, or a value of 2.83 (see Table 1). Given that the material is exposed to gaseous hydrogen above some threshold value, the literature suggests that the products *m1*·*d1* and *m2*·*d2* can be as low as 0.1 and as high as 1.2 [8,9]. The upper bound for *B1* and *B2* is difficult to quantify and may be based on the *ΔK* at which stage III fast fracture occurs (approaching infinity). As *B1* and *B2* have the greatest effect on the predicted transition crack length, and given that the transition crack length is proportional to the FPZ size (six times the FPZ size in the case of X100 [1]), the “correct” value of *B1* in conjunction with *B2* may be selected by ensuring a transition crack length that is reasonable for the material given its predicted FPZ size.

When appropriate values for all parameters are established, reasonable ranges beyond the baseline values can be established based upon typical pipeline operating conditions. Pressure in a natural gas pipeline routinely fluctuates up to ±10 % on any given day [10]. A hydrogen pipeline can be expected to operate at pressures of approximately 10.3 MPa. The pipeline pressure could therefore be expected to fluctuate between 9.3 MPa and 11.3 MPa every day. If the parameter *m1* is varied from its original value (0.25) to any value between 0.239 and 0.260 at normal operating pressure, the consequence to the model will be equivalent to that of a routine pressure fluctuation in the pipeline. Similarly, if *m2* is varied from its original value of 0.22 to any value between 0.210 and 0.229 at normal operating pressure, the change will correspond to a routine pressure fluctuation.

The stress ratio *R_σ_* in a liquid pipeline routinely ranges from 0.6 to 0.8 [11]. The reasonable range of *B1* and *B2* depends on the *ΔK* and the maximum or minimum value of *K* (*K_min_* or *K_max_*) experienced by the pipeline. For instance, a *ΔK* of 6 MPa·m^1/2^ and an *R_σ_* of 0.7 corresponds to a *Kmin* of 14 MPa·m^1/2^. Variation in *B1* between 5.565 and 9.923 at *ΔK* = 6 MPa·m^1/2^ and *K_min_* = 14 MPa·m^1/2^ corresponds to fluctuations in *K_max_* that could be expected, given routine fluctuations in stress ratio. Similarly, *ΔK* = 30 MPa·m^1/2^ and *R_σ_* = 0.7 corresponds to *K_min_* = 70 MPa·m^1/2^. Variation of *B2* between 2.668 and 3.582 at *ΔK* = 30 MPa·m^1/2^ and *K_min_* = 70 MPa·m^1/2^ corresponds to routine fluctuations in *K_max_*.

## 3. Conclusions

The conclusions that can be drawn based upon the sensitivity analysis of the HA FCG model parameters are as follows:

1. The exponents on the stress intensity range *ΔK*, *B1* and *B2*, have far more impact on the predicted FCG response than do *m1* and *m2*, the exponents on the hydrogen pressure term.
  - a. Modifying *B1* and *B2* has the effect of shifting the predicted FCG response up and down, thereby greatly affecting the transition crack length separating the transient and steady state responses. The values of *B1* and *B2* must be selected with an eye towards the FPZ size and the effects it has upon the transition crack length.
2. The exponent acting upon the hydrogen pressure in the transient regime, *m1*, generally shifts the predicted HA FCG response left and right. This modification has little effect upon the predicted transition crack length. As such, the value of *m1* may be determined by matching the *da/dN* and *ΔK* value at which the HA FCG response deviates from that of air (*ΔK* values in the range of 6–9 MPa·m^1/2^).
3. Although modifying the exponent acting upon the hydrogen pressure in the steady state regime, *m2*, shifts the predicted response up and down, the effect is primarily far from the transition crack length, and research has indicated that the FCG response of API steels is minimally affected (if at all) by the magnitude of hydrogen pressure in this FCG regime [12]. As such, the authors conclude that this parameter value can be set to that of air as a first-order estimate.

## Figures and Tables

**Table 1:** Parameter values for full model calibration to API X100 [1]

API-5L X100			
A	9.9×10^−9^	b	2.83
Transient		Steady State	
a1	1.5×10^−4^	a2	1.3×10^−4^
B1	7.96	B2	3.17
m1	0.25	m2	0.22
d1	3	d2	1
